# High Sodium and Low Potassium Intake among Italian Children: Relationship with Age, Body Mass and Blood Pressure

**DOI:** 10.1371/journal.pone.0121183

**Published:** 2015-04-08

**Authors:** Angelo Campanozzi, Sonia Avallone, Antonio Barbato, Roberto Iacone, Ornella Russo, Gianpaolo De Filippo, Giuseppina D’Angelo, Licia Pensabene, Basilio Malamisura, Gaetano Cecere, Maria Micillo, Ruggiero Francavilla, Anna Tetro, Giuliano Lombardi, Lisa Tonelli, Giuseppe Castellucci, Luigi Ferraro, Rita Di Biase, Antonella Lezo, Silvia Salvatore, Silvia Paoletti, Alfonso Siani, Daniela Galeone, Pasquale Strazzullo, MINISAL-GIRCSI Program Study Group

**Affiliations:** 1 Pediatrics, University of Foggia, Foggia, Italy; 2 Department of Clinical Medicine and Surgery, Federico II University of Naples Medical School, ESH Excellence Centre of Hypertension, Naples, Italy; 3 Assistance Publique—Hôpitaux de Paris, Service d’Endocrinologie et Diabétologie Pédiatrique, Hôpital Bicêtre, Le Kremlin-Bicêtre, France; 4 Pediatrics, University Magna Graecia, Catanzaro, Italy; 5 Pediatrics Hospital of Cava dei tirreni (SA), Italy; 6 Pediatrics, ASL-Naples, Italy; 7 Pediatrics, University of Bari, Bari, Italy; 8 Pediatrics, San Paolo Hospital, Bari, Italy; 9 Pediatrics, Santo Spirito Hospital, Pescara, Italy; 10 Pediatrics, University of Marche, Ancona, Italy; 11 Pediatrics, ASL Umbria, Perugia, Italy; 12 Pediatrics, University of Modena, Modena, Italy; 13 Pediatrics, University of Torino, Italy; 14 Pediatrics, University of Insubria, Varese, Italy; 15 Pediatrics, ASL-Roma, Rome, Italy; 16 Epidemiology & Population Genetics, Institute of Food Science & Technology, National Research Council, Avellino, Italy (AS); 17 Italian Ministry of Health, Center for Disease Prevention and Control, Rome, Italy; University of Florida, UNITED STATES

## Abstract

**Background:**

Hypertension is the leading cause of death in developed countries and reduction of salt intake is recommended as a key preventive measure.

**Objective:**

To assess the dietary sodium and potassium intakes in a national sample of Italian children and adolescents and to examine their relationships with BMI and blood pressure (BP) in the framework of the MINISAL survey, a program supported by the Italian Ministry of Health.

**Population and Methods:**

The study population included 1424 healthy subjects (766 boys, 658 girls) aged 6-18 years (mean age: 10.1±2.9) who were consecutively recruited in participating National Health Service centers in 10 Italian regions. Electrolyte intake was estimated from 24 hour urine collections tested for completeness by the concomitant measurement of creatinine content. Anthropometric indices and BP were measured with standardized procedures.

**Results:**

The average estimated sodium intake was 129 mmol (7.4 g of salt) per day among boys and 117 mmol (6.7 g of salt) among girls. Ninety-three percent of the boys and 89% of the girls had a consumption higher than the recommended age-specific standard dietary target. The estimated average daily potassium intakes were 39 mmol (1.53 g) and 36 mmol (1.40 g), respectively, over 96% of the boys and 98% of the girls having a potassium intake lower than the recommended adequate intake. The mean sodium/potassium ratio was similar among boys and girls (3.5 and 3.4, respectively) and over 3-fold greater than the desirable level. Sodium intake was directly related to age, body mass and BP in the whole population.

**Conclusions:**

The Italian pediatric population is characterized by excessive sodium and deficient potassium intake. These data suggest that future campaigns should focus on children and adolescents as a major target in the framework of a population strategy of cardiovascular prevention.

## Introduction

Excess sodium and inadequate potassium intake have detrimental effects on blood pressure (BP) [1–3] and are both associated with increased risk of stroke, cardiovascular risk and premature death [4–7]. Adequate sodium and potassium intake is one of the life style modifications currently recommended for prevention and treatment of hypertension and cardiovascular disease [8].

As dietary habits are developed during childhood [9], including the preference for salted foods [10, 11], education to keep a low dietary salt and an adequate potassium intake during childhood is crucial. We report here on the estimated dietary sodium and potassium intake, the sodium to potassium ratio and their relationship to age, body mass and BP in a national sample of Italian children and adolescents examined in the framework of the MINISAL-GIRCSI Program [12,13] with the collaboration of the Italian Society for Pediatric Gastroenterology, Hepatology and Nutrition (SIGENP).

## Methods

### Study population

The participants’ recruitment was organized by the Italian Society for Pediatric Gastroenterology, Hepatology and Nutrition (SIGENP) regional coordinators and materially operated by the pediatricians and the general practitioners. The participating pediatricians and general practitioners were thoroughly informed about the project and were asked to consecutively recruit, among their patients, healthy subjects aged 6–18 years who would be willing to go through the study procedures and whose parents or legal tutors would give their informed consent to the study.

### Study procedures

These involved the administration of a questionnaire concerning family and personal history, habitual physical activity and dietary habits, a standard physical examination and an anthropometric evaluation (height, weight, body-mass index). BMI was calculated for each subject and BMI z-score was assessed, according to Centers for Disease Control and prevention (CDC) growth charts (http://www.cdc.gov/nchs/data/series/sr_11/sr11_246.pdf) [14].

At the end of the visit, the participants (or their caregivers in the case of younger children) received a plastic container for 24h urine samples together with detailed oral and written instructions on how to collect complete 24h urines, as previously described [13]. Once the collection was returned, the subject was required to confirm completeness of the collection, the total urine volume was recorded and two samples were extracted, immediately stored in plastic containers and frozen at -30°C to be later analysed by the central laboratory at Federico II University of Naples, as previously reported [13]. 24 h urinary excretions were used as proxies for the respective dietary intakes, according to WHO recommendation [15]. Urinary sodium and potassium concentrations were measured by ion selective electrode potentiometry and urinary creatinine by a kinetic Jaffe′ reaction using an ABX Pentra 400 apparatus (HORIBA ABX, Rome, Italy). Quality control was performed using urine specific reference samples from UrichemGol BIO-DEV (Milan, Italy). The inter-assay technical error was 0.73% for sodium, 1.16% for potassium and 1.12% for creatinine.

### Statistical analysis

Statistical analysis was performed using the Statistical Package for the Social Sciences (SPSS-PC version 13, SPSS Inc., Chicago, Illinois, USA) and was aimed at the assessment of the differences occurring in sodium and potassium intake by gender and by age-related pubertal stage.

Thus, separate analyses were conducted for male and female participants. Assuming a normal age at onset of puberty after 8 years for girls and 9 years for boys, and a mean age of children at Tanner stage 2 of 10.2 years for girls and 11.5 years for boys [16], the two sub-populations were stratified in three groups each and referred to as pre-pubertal children (boys < 9, girls < 8 years), peri-pubertal children (boys 9 to 11.5, girls 8 to 10.2 years) and adolescents (boys > 11.5, girls > 10.2 years). In other analyses, the two sub-populations were stratified by geographic location (Southern, Central and Northern Italy).

As most variables did not have a Gaussian distribution as assessed by the test of Kolmogorov-Sminorf (SPSS-PC version 13), non-parametric tests were used for the assessment of differences in group means (Kruskal-Wallis). Spearman rank correlation analysis was used to evaluate the possible associations between sodium or potassium intake and systolic blood pressure. Given potential confounding by age and BMI on these associations, multiple linear regression analysis was used to evaluate the relative influences of age, body mass and estimated sodium or potassium intake (using as proxies their respective 24h urinary excretion) on systolic BP. The analyses were carried out on the whole study population as well as separately in boys and girls. Various models were implemented with the stepwise addition to the equation of the various factors investigated. Given the high degree of inter-correlation between urinary sodium and potassium excretion, these two variables were always added to separate models. The Z-scores of the variables under investigation were used in all these analyses.

The results were expressed as median and interquartile range, unless otherwise indicated. Two sided p-values less than 0.05 were considered statistically significant.

The study protocol was approved by the scientific committee of the Ministry of Health and by Italian Society for Pediatric Gastroenterology, Hepatology and Nutrition (SIGENP). Written informed consent for participating in the study was obtained from survey participants and their parents.

## Results

A total of 1625 subjects aged 6–18 years were enrolled (86.1% of those invited) in 10 italian regions representative of the whole Italian territory (*Northern Italy*: Piemonte, Lombardia, Emilia Romagna; *Central Italy*; Toscana, Umbria, Marche, Lazio; *Southern Italy*: Puglia, Campania, Calabria) and provided a 24-h urine collection. Two-hundred-one subjects were excluded from the analysis because of the suspicion of incomplete 24h urine collection as their urinary creatinine level referred to body weight was lower than 0.1 mmol/kg body weight per day, a value identified in a previous validation study [17] as the fifth percentile of the 24h urinary creatinine distribution in a population of healthy individuals aged 3 to 18 years.

Thus, the study population was made of 1424 healthy children and adolescents (mean age: 10.1±2.9 years): 766 boys (mean age 10.1 years, 95% CI = 9.9–10.3) and 658 girls (10.4 years, 95% CI = 10.1–10.6). Gender-specific median values and interquartile range for urine volume, creatinine, sodium and potassium excretion in the whole population are given in Table 1. Whereas 24-hour urine volume was similar in boys and girls, the boys had significantly higher urinary sodium (p = 0.001), slightly higher urinary potassium (p = 0.003) and also higher creatinine excretion (p = 0.001) than girls. The mean urinary sodium/potassium ratio was similar in boys and girls (3.2, IQ = 3.1–3.3, vs 3.1, IQ = 3.0–3.3, p = NS).

**Table 1:** Gender specific 24h sodium, potassium, creatinine urinary excretion and urinary volume according to stage of sexual development.

Boys (n = 766)				
	Sodium mmol/24h	Potassium mmol/24h	Creatinine mmol/24h	Urine volumeml/24h
Age	Median(interquartile range)	Median (interquartile range)	Median (interquartile range)	Median (interquartile range)
**All**	**120 (84–162)**	**37 (27–49)**	**6.6 (4.6–9.2)**	**900 (700–1200)**
**<9 yrs (n = 303)Pre-pubertal**	**98 (73–130)**	**33 (25–44)**	**4.9 (3.8–6.5)**	**800 (600–1000)**
**9 yrs-11 yrsand 5 months(n = 228) Peri-pubertal**	**124 (94–168)**	**37 (29–49)**	**6.8 (5.4–8.6)**	**900 (700–1250)**
**≥11 yrs and 5months(n = 235) Adolescence**	**136 (102–192)**	**42 (30–54)**	**9.6 (6.9–13.7)**	**1100 (800–1410)**
**p for trend**	**<0.001**	**<0.001**	**<0.001**	**<0.001**
**Girls (n = 658)**				
	**Sodium mmol/24h**	**Potassium mmol/24h**	**Creatinine mmol/24h**	**Urine volumeml/24h**
**Age**	**Median(interquartile range)**	**Median (interquartile range)**	**Median (interquartile range)**	**Median (interquartile range)**
**All**	**107 (77–146)**	**34 (26–44)**	**5.8 (4.1–8.1)**	**900 (700–1205)**
**<8 yrs(n = 162) Pre-pubertal**	**91 (65–118)**	**29 (23–39)**	**4.2 (3.3–5.6)**	**750 (600–950)**
**8–10 yrs and 2months(n = 180) Peri-pubertal**	**98 (68–125)**	**33 (25–40)**	**5.0 (3.8–6.5)**	**850 (650–1200)**
**≥10 yrs and 2months(n = 316) Adolescence**	**126 (93–172)**	**39 (28–49)**	**7.6 (5.6–9.9)**	**1050 (800–1400)**
**p for trend**	**<0.001**	**<0.001**	**<0.001**	**<0.001**

**Fig 1:**
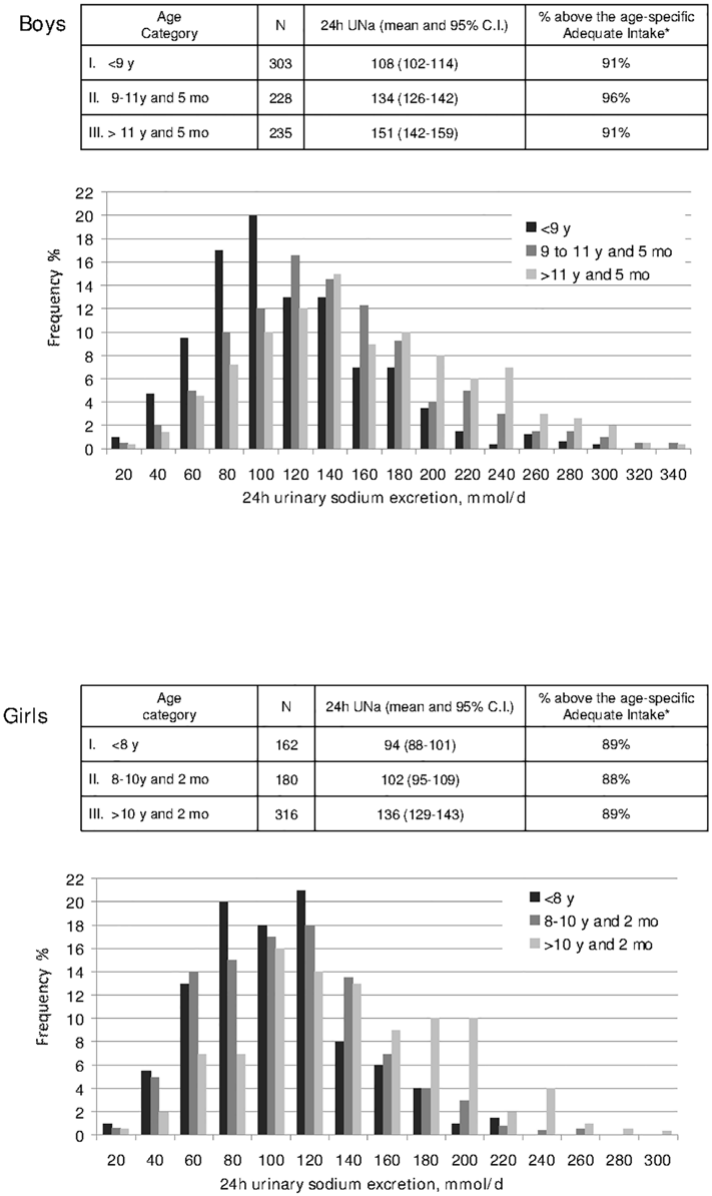
Gender-specific frequency distribution of 24-hour urinary sodium excretion in boys (A) and girls (B) (data from 10 Italian regions, age 6–18 years, MINISAL-GIRCSI Program). The insets in the figures report the mean values of electrolyte excretion by development stage and denote the percentage of individuals complying with the respective adequate intakes as established by the Italian Society for Human Nutrition and the Institute for Food Research and Nutrition (ref. 18). *Data based on the assumption that 24h urinary sodium excretion provides an estimate of daily sodium intake.

**Fig 2:**
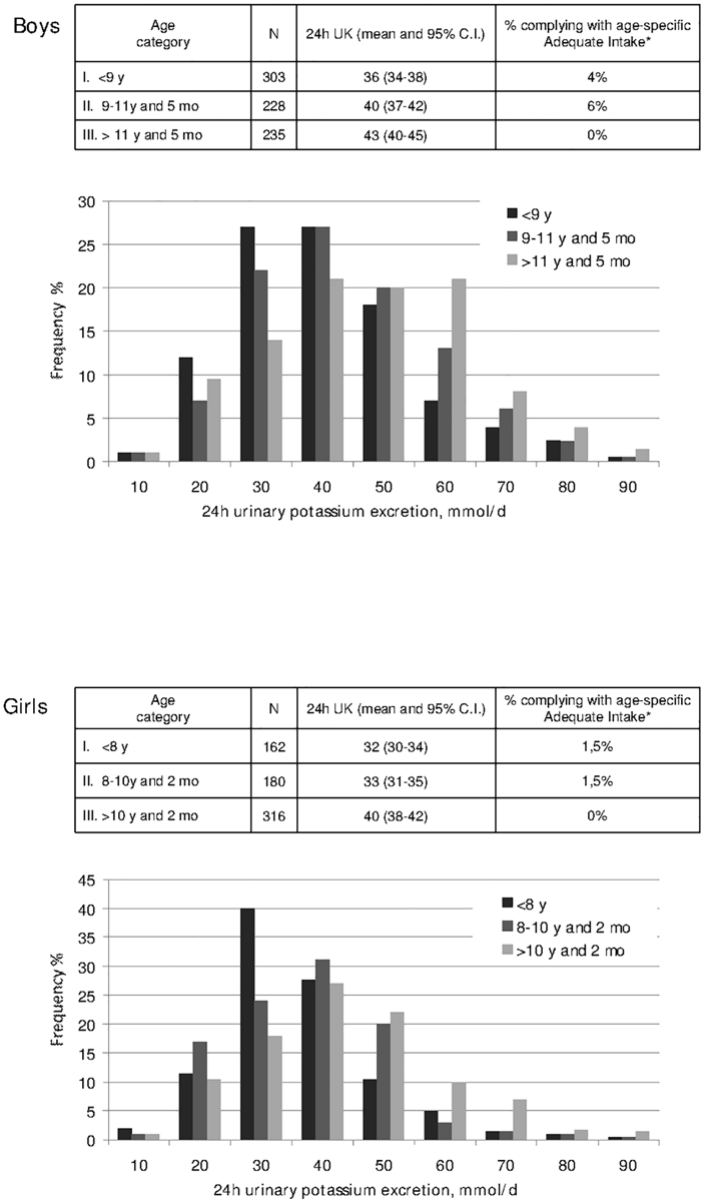
Gender-specific frequency distribution of 24-hour urinary potassium excretion in boys (A) and girls (B) (data from 10 Italian regions, age 6–18 years, MINISAL-GIRCSI Program). The insets in the figures report the mean values of electrolyte excretion by development stage and denote the percentage of individuals complying with the respective adequate intakes as established by the Italian Society for Human Nutrition and the Institute for Food Research and Nutrition (ref. 18). *Data based on the assumption that 24h urinary sodium excretion provides an estimate of daily potassium intake.

Figs. 1A - 1B and 2A - 2B display the frequency distribution of urinary sodium and potassium excretion by stage of estimated sexual development. A stepwise shift-to-the-right in the frequency distributions of both sodium and potassium excretion was apparent with advancing sexual development. As shown in the figure insets, over 90% of the participants at any developmental stage had a 24h urinary sodium excretion indicating an intake well above the standard dietary target recently set by the Italian Society of Human Nutrition and the Institute of Food Research and Nutrition [18]. Likewise, less than 3% of the participants appeared to be compliant with the recommended intake for potassium at any developmental stage.

Table 1 also reports the 24h urine volume and the sodium, potassium and creatinine excretion in boys and girls upon stratification by stage of sexual development. For all the four variables, a highly significant trend was apparent to a progressive increase with age (p<0.001) and more so in the transition from the peri-pubertal age to adolescence. Creatinine excretion approximately doubled between prepubertal and adolescent subjects, sodium excretion increased by 39% in both boys and girls whereas potassium intake increased by 19% and 21%, respectively. As reported in S1 Table, there was no clear cut geographical trend for either sodium or potassium excretion in both boys and girls.

**Fig 3:**
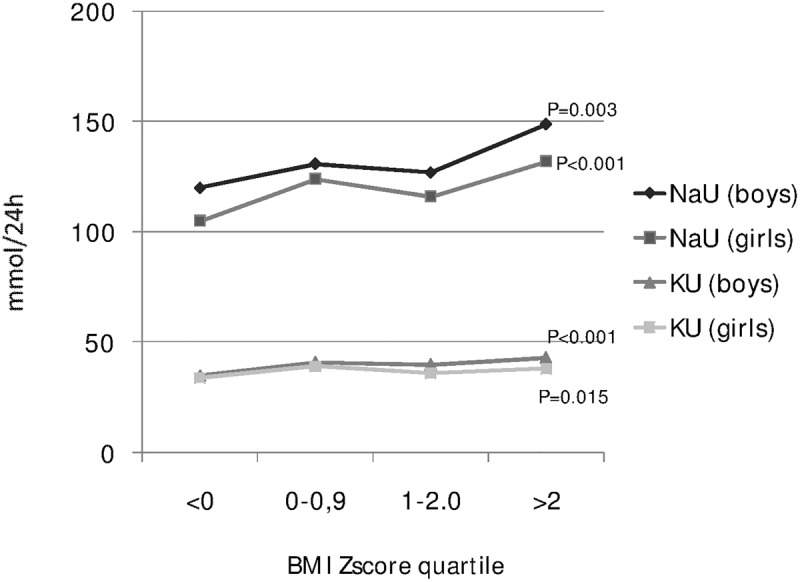
Gender-specific association between body mass index and 24-hour urinary sodium and potassium excretion (data from 10 Italian regions, age 6–18 years, MINISAL-GIRCSI Program).

The gender-specific association between body mass index and 24-hour urinary sodium and potassium excretion is shown in Table 2 and Fig. 3. Twenty-four hour urinary sodium excretion and, to a lower extent, urinary potassium excretion were directly related to age and BMI-Z-score in simple linear correlation analysis (Table 3). In this same analysis, systolic BP (expressed as z-score) was also directly related to 24h urinary sodium and potassium excretion (Table 3). An exhaustive representation of multiple linear regression analysis of these associations accounting for potential confounding by age and body mass is given by the summary models reported in Table 4. Overall, age and body mass were the two main determinants of systolic BP in the whole population as well as separately in boys and girls. Twenty-four hour urinary sodium excretion was weakly related to systolic BP Z-score in the whole population, but not in separate analyses of boys and girls, even upon adjustment for age and BMI.

**Table 2:** Gender specific 24h sodium, potassium, creatinine urinary excretion and urinary volume according to BMI-Zscore.

Boys (n = 766)				
	Sodium mmol/24h	Potassium mmol/24h	Creatinine mmol/24h	Urine volumeml/24h
BMI Zscore	Median (interquartile range)	Median (interquartile range)	Median (interquartile range)	Median (interquartile range)
< 0 (n = 208)	111 (80–150)	33 (25–43)	6.0 (4.3–8.5)	850 (602–1150)
0–0.9 (n = 234)	124 (89–160)	38 (28–51)	6.4 (4.6–9.4)	950 (787–1252)
1–2 (n = 240)	114 (81–169)	37 (27–50)	6.9 (4.6–9.3)	850 (650–1172)
> 2 (n = 84)	135 (93–198)	42 (32–53)	7.1 (5.4–9.4)	975 (762–1290)
p for trend	0.010	<0.001	0.068	0.006
Girls (n = 658)				
	Sodium mmol/24h	Potassium mmol/24h	Creatinine mmol/24h	Urine volumeml/24h
BMI Zscore	Median (interquartile range)	Median (interquartile range)	Median (interquartile range)	Median(interquartile range)
< 0 (n = 190)	98 (72–131)	31 (23–41)	5.4 (3.7–7.8)	850 (700–1200)
0–0.9 (n = 202)	114 (88–153)	36 (27–46)	6.3 (4.5–8.7)	920 (730–1220)
1–2 (n = 216)	108 (73–148)	34 (24–43)	5.6 (4.1–8.1)	900 (650–1300)
> 2 (n = 50)	116 (90–159)	35 (28–45)	6.3 (3.8–8.2)	900 (737–1225)
p for trend	0.002	0.008	0.031	0.308

**Table 3:** Gender-specific simple linear correlation analysis: 24h urinary sodium and potassium excretion and specified variables (Spearman correlation coefficients rS).

Boys (n = 766)								
	24h urinary sodium excretion				24h urinary potassium excretion			
	Age	BMI	BMI Z-score	SystolicBP	Age	BMI	BMI Z-score	SystolicBP
All	0.327**	0.272**	0.088*	0.155**	0.196**	0.252**	0.139**	0.155**
<9 yrs(n = 303) Pre-pubertal	0.170**	0.119*	0.090	-0.013	-0.003	0.091	0.090	-0.006
9 yrs-11 yrsand 5 months (n = 228) Peri-pubertal	0.097	0.236**	0.221**	0.121	0.046	0.392**	0.306**	0.180**
≥11 yrs and 5months(n = 235) Adolescence	0.152*	0.217**	0.159*	0.040	0.128	0.224**	0.172**	0.107
Girls (n = 658)								
	24h urinary sodium excretion				24h urinary potassium excretion			
	Age	BMI	BMI Z-score	Systolic BP	Age	BMI	BMI Z-score	SystolicBP
All	0.314**	0.260**	0.090*	0.220**	0.240**	0.177**	0.042	0.187**
<9 yrs(n = 162) Pre-pubertal	-0.029	0.167*	0.182*	0.019	0.026	0.019	0.030	0.006
9 yrs-11 yrsand 5 months(n = 180) Peri-pubertal	0.043	0.136	0.130	0.149*	0.140	0.120	0.096	0.180*
≥11 yrs and 5months(n = 316) Adolescence	0.084	0.217**	0.191**	0.144*	0.072	0.158**	0.124*	0.120*

** Significant at the 0.001 level (2-tailed)

* Significant at the 0.05 level (2-tailed)

**Table 4:** Multiple linear regression analysis of systolic BP on age, BMI and 24h urinary sodium or potassium excretion.

Whole population (n = 1424)								
	Model A				Model B			
	B	95% C.I.	T	p	B	95% C.I.	T	p
Z-Age	0.269	0.219–0.319	10.46	<0.001	0.276	0.226–0.325	10.94	<0.001
Z-BMI	0.266	0.216–0.316	10.44	<0.001	0.270	0.220–0.319	10.70	<0.001
Z-24h sodium excretion	0.056	0.006–0.105	2.19	0.028	-	-	-	-
Z-24h potassium excretion	-	-	-	-	0.050	0.003–0.098	2.07	0.038
Boys (n = 766)								
	Model A				Model B			
	B	95% C.I.	T	p	B	95% C.I.	T	p
Z-Age	0.301	0.230–0.372	8.29	<0.001	0.307	0.237–0.376	8.65	<0.001
Z-BMI	0.260	0.188–0.332	7.05	<0.001	0.260	0.188–0.332	7.11	<0.001
Z-24h sodium excretion	0.042	-0.026–0.109	1.21	0.225	-	-	-	-
Z-24h potassium excretion	-	-	-	-	0.045	-0.019–0.109	1.39	0.165
Girls (n = 658)								
	Model A				Model B			
	B	95% C.I.	T	p	B	95% C.I.	T	p
Z-Age	0.232	0.160–0.303	6.36	<0.001	0.240	0.169–0.310	6.68	<0.001
Z-BMI	0.273	0.204–0.342	7.78	<0.001	0.281	0.213–0.349	8.10	<0.001
Z-24h sodium excretion	0.076	0.00–0.151	1.97	0.050	-	-	-	-
Z-24h potassium excretion	-	-	-	-	0.060	-0.013–0.133	1.62	0.105

B = standardised regression coefficient, T = t-test

## Discussion

### Salt consumption by Italian children and adolescents

The data presented herewith from a national sample of boys and girls aged 6 to 18 years indicate that the average daily sodium consumption in Italy exceeds the official recommendations in both genders, in all age categories and, in all regions. Boys had a higher sodium intake than girls probably because of a higher food intake. Based on the recently released national recommendations [18], the age- specific standard dietary targets for sodium intake (1.5 g/d between age 7 and 10 and 2 g/d above 10 yrs) were exceeded by 93% of the boys and by 89% of the girls. The sodium intake levels of Italian children and adolescents are comparable to those recently reported by the New Zealand 2003–2004 Total Diet Survey, which however was based on dietary recalls and thus tended to underestimate true sodium consumption [19]. The Italian sodium intake levels are also somewhat lower compared with those reported by the National Health and Examination Survey (NHANES) 2003–2006 for a U.S population in the same age-range [20], again using dietary questionnaires most likely underestimating true sodium intake.

The detection of significant differences in sodium intake between boys and girls in our study population at all stages of sexual development suggests that sex hormones do not play a major role per se in the trend to progressively greater salt intake from childhood to adolescence. Conversely, this between-gender difference, together with the direct association observed between 24h sodium excretion and age, suggests that higher sodium intake is a consequence of the increased food consumption. The very high correlation coefficients (0.7 to 0.8) between sodium and energy intake and the similarity of the sodium/energy ratio between male and female subjects shown in previous reports [20] further support this interpretation. The finding of a gradual increase in estimated sodium intake with increasing BMI Z-score in the MINISAL young population argues for an additive contribution of adiposity to greater sodium consumption, similarly to what observed in the recent MINISAL survey of a sample of Italian adult population [13]. These results are worrying especially when considering that overweight in adolescents is associated with higher salt-sensitivity of BP [21]. Moreover, higher salt intake in this age-group is associated with greater consumption of sugar-sweetened high calorie soft drinks, which in turn is expected to favor fat accumulation [22, 23].

Salt intake was weakly associated with BP Z-score at multiple linear regression analysis in the whole study population upon accounting for the effects of age and body mass but this association was no longer statistically significant in the gender-specific analyses. These results are not surprising as the detection of true biological correlations between habitual sodium intake and any potentially related variable, including BP, is severely hampered by the large regression dilution bias affecting the estimate of dietary salt intake through a single 24h urine collection [24]. Moreover, the effects of sodium intake on BP are expected to become progressively greater and measurable with advancing age. In fact, in a recent survey, high blood pressure was detected in 5–10% of Italian adolescents [25].

### Potassium consumption among children and adolescents in Italy

Daily potassium intake in the MINISAL young population was much lower than the age-specific adequate intakes (2800 mg/d between age 7 and 10 and 3900 mg/d above age 10 yrs) in both boys and girls, in all the age categories and in all the regions surveyed. Based on the Italian national recommendations, over 96% of the boys and 98% of the girls had a potassium intake lower than the age-specific adequate intake. These percentages are similar to those found by the recent MINISAL survey of a national sample of adult population [13], suggesting that the largely inadequate potassium consumption in Italy might derive from negative dietary habits acquired early in life [9, 10]. Inadequate potassium intake was found also in other young populations worldwide, e.g. among 7–10 year-old British children [26], among French children [27] and in U.S. young population groups [28].

Similarly as for sodium intake, the estimated potassium intake was directly associated with age and body mass but its rate of increase from the pre-pubertal stage to adolescence was much lower (19% increase in potassium intake from pre- to post-pubertal age among boys and 25% among girls) compared with the parallel increase in sodium intake (40% increase among boys and 45% among girls). This observation suggests that, although potassium similarly to sodium consumption follows the rise in food and calorie intake with age, nevertheless in the shift from childhood to adolescence an increasing preference is acquired for salty foods as compared with that for potassium-rich natural foods. In an Icelandic population of 6 year-old children higher sodium intake was associated with poorer quality of diet while the opposite was true for potassium intake [29]. In an analysis of the NHANES 1999–2002 database, the proportion of children and adolescents who reached the age-specific adequate intake level for potassium was six-fold higher among those who met the Food Guide System dairy recommendations than among those who did not [28].

Although 24h-urinary potassium excretion was related to BP in simple linear correlation analysis, no independent association of potassium intake with systolic BP was observed at multivariable analysis. A high-potassium diet was shown to lower BP in both hypertensive and normotensive adult individuals [30], whereas the results of observational and intervention studies of potassium intake and BP in youth did not provide consistent results [31]. In the present study, we assessed this possible association at a very young age, when the possible effects of reduced potassium intake may not yet be apparent. Furthermore, it should be noted that almost all the participants surveyed did not meet the national recommendations for potassium intake, this making it difficult to assess the possible effect of an adequate intake of this electrolyte on BP.

## Strengths and Limitations

This is the first survey of sodium and potassium intake in a national sample of children and adolescents in Italy. A major strength of our study is the use of 24h urine collections for the assessment of dietary sodium and potassium intake, following WHO recommendations [15]. The response rate was very high (86.1%). The 24h urine collection captures 90% or more of the ingested sodium and potassium and is unaffected by the recognised limitations of dietary food recalls or dietary interviews. The use of urinary creatinine excretion as a marker of completeness of the urine collection provided reasonable confidence in the accuracy of the samples included in the analysis. Finally, another strength of the study was the centralized measurement of the biochemical variables thus keeping the measurement variability to a tight quality control system.

However, there were several methodological limitations. The MINISAL study did not recruit a truly statistical sample of the children and adolescent population of the regions participating in the survey nor was the survey extended to all the Italian regions. Another study limitation is the misclassification of the “individual” electrolyte intake due to the availability of a single 24h urine collection. Indeed, for the assessment of the true biological associations occurring between sodium or potassium intake and any other variable under investigation, including BP, multiple 24h urine collections would be necessary and these should be possibly combined with the results of an effective dietary evaluation [1]. The individual electrolyte intake, however, was not a primary objective of the MINISAL study. Finally, the classification in prepubertal, peripubertal and pubertal status, in the absence of a complete medical evaluation due to the constraints of an epidemiological design, was to some extent arbitrary.

## Conclusions

In conclusion, the results of the MINISAL survey of a large sample of children and adolescents in the age range 6 to 18 years from 10 Italian regions indicate that the habitual sodium intake was well above the age-specific standard dietary target and the potassium intake dramatically below the adequate intake recommended for the Italian population. These results add compelling evidence of the urgent need to implement a systematic strategy for the reduction of dietary sodium intake at the population level in Italy.

The MINISAL results also suggest that the largely inadequate levels of potassium intake recently documented in the Italian adult population [13] might have their roots in early childhood, calling for a tighter collaboration among the Italian pediatricians, the school education system and the children’s parents in the effort to reduce the gap between the current negative features of Italian dietary habits and the classical model of Mediterranean diet featuring generous consumption of natural foods such as fruits and vegetables and a watchful attitude against the current abuse of low quality high energy salt-rich foods starting in childhood.

These data indicate that future campaigns, aiming at increasing the awareness about correct sodium and potassium intakes, should focus on children and adolescents as a major target in the framework of a population strategy of cardiovascular prevention.

## Supporting Information

S1 Table Gender-specific 24h urinary sodium and potassium excretion by geographical area. (TIF)
