# Supplementary figures and images for: High Sodium and Low Potassium Intake among Italian Children: Relationship with Age, Body Mass and Blood Pressure
Source: PLoS One. 2015 Apr 8;10(4):e0121183. doi: 10.1371/journal.pone.0121183 (PMC4390153; doi:10.1371/journal.pone.0121183)

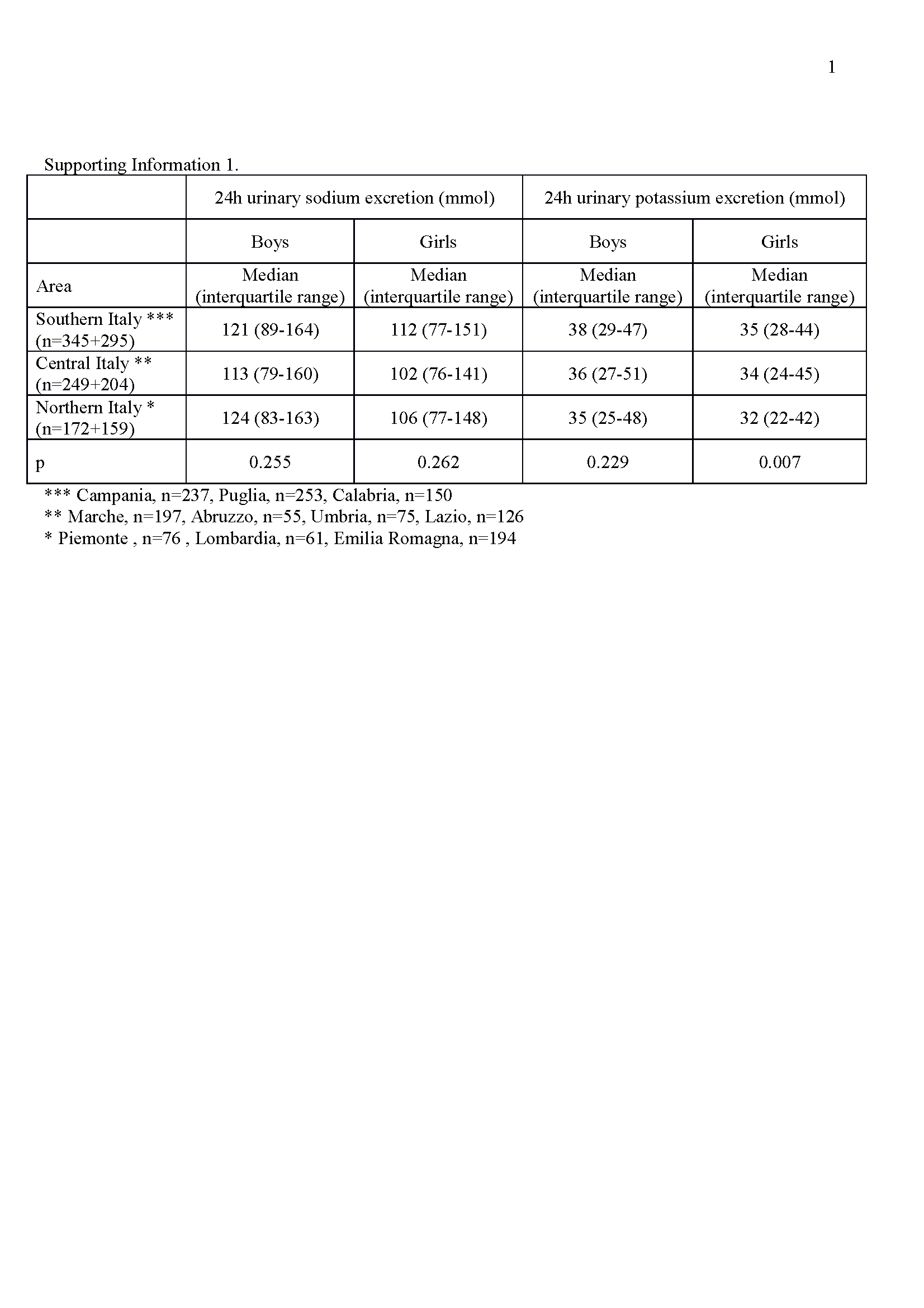

Supplement: S1 Table — (TIF) [file pone.0121183.s001.tif]
